# Increasing uptake of structured self-management education programmes for type 2 diabetes in a primary care setting: a feasibility study

**DOI:** 10.1186/s40814-020-00606-0

**Published:** 2020-05-22

**Authors:** Melanie Davies, Caroline A. Kristunas, Lisa Huddlestone, Abualbishr Alshreef, Danielle Bodicoat, Simon Dixon, Helen Eborall, Agnieszka Glab, Nicky Hudson, Kamlesh Khunti, Graham Martin, Alison Northern, Mike Patterson, Rebecca Pritchard, Sally Schreder, Bernie Stribling, Jessica Turner, Laura J. Gray

**Affiliations:** 1 Diabetes Research Centre, University of Leicester, Leicester, UK; 2 Department of Health Sciences, University of Leicester, Leicester, UK; 3 School of Health and Related Research, University of Sheffield, Sheffield, UK; 4 Leicester Diabetes Centre, University Hospitals of Leicester NHS Trust, Leicester, UK; 5 Centre for Reproduction Research, School of Applied Social Science, De Montfort University, Leicester, UK; 6 THIS Institute, University of Cambridge, Cambridge, UK

**Keywords:** Type 2 diabetes, Structured self-management education, Feasibility study

## Abstract

**Background:**

Structured self-management education (SSME) for people with type 2 diabetes mellitus (T2DM) improves biomedical and psychological outcomes, whilst being cost-effective. Yet uptake in the UK remains low. An ‘Embedding Package’ addressing barriers and enablers to uptake at patient, health care professional and organisational levels has been developed. The aim of this study was to test the feasibility of conducting a subsequent randomised controlled trial (RCT) to evaluate the Embedding Package in primary care, using a mixed methods approach.

**Methods:**

A concurrent mixed methods approach was adopted**.** Six general practices in the UK were recruited and received the intervention (the Embedding Package). Pseudonymised demographic, biomedical and SSME data were extracted from primary care medical records for patients recorded as having a diagnosis of T2DM. Descriptive statistics assessed quantitative data completeness and accuracy. Quantitative data were supplemented and validated by a patient questionnaire, for which two recruitment methods were trialled. Where consent was given, the questionnaire and primary care data were linked and compared. The cost of the intervention was estimated. An integrated qualitative study comprising ethnography and stakeholder and patient interviews explored the process of implementation, sustainability of change and ‘fit’ of the intervention. Qualitative data were analysed using a thematic framework guided by the Normalisation Process Theory (NPT).

**Results:**

Primary care data were extracted for 2877 patients. The primary outcome for the RCT, HbA1c, was over 90% complete. Questionnaires were received from 423 (14.7%) participants, with postal invitations yielding more participants than general practitioner (GP) prompts. Ninety-one percent of questionnaire participants consented to data linkage. The mean cost per patient for the Embedding Package was £8.94, over a median follow-up of 162.5 days. Removing the development cost, this reduces to £5.47 per patient. Adoption of ethnographic and interview methods in the collection of data was appropriate, and the use of NPT, whilst challenging, enhanced the understanding of the implementation process. The need to delay the collection of patient interview data to enable the intervention to inform patient care was highlighted.

**Conclusions:**

It is feasible to collect data with reasonable completeness and accuracy for the subsequent RCT, although refinement to improve the quality of the data collected will be undertaken. Based on resource use data collected, it was feasible to produce cost estimates for each individual component of the Embedding Package. The methods chosen to generate, collect and analyse qualitative data were satisfactory, keeping participant burden low and providing insight into potential refinements of the Embedding Package and customisation of the methods for the RCT.

**Trial registration:**

ISRCTN, ISRCTN21321635, Registered 07/07/2017—retrospectively registered.

## Key messages regarding feasibility

- The feasibility of data collection procedures and utility of the chosen theoretical framework were uncertain.
- The study identified that it was feasible to collect quantitative data with reasonable completeness and accuracy directly from primary care. The collection of qualitative data was also found to be feasible, and the appropriateness of the selected theoretical framework was confirmed.
- Feasibility and potential benefits of the intervention were indicated. Refinement of quantitative data collection methods was recommended to enhance quality. Further development of the Embedding Package was highlighted to improve clinician engagement.

## Background

Type 2 diabetes mellitus (T2DM) is a progressive chronic disease which can lead to a reduced quality of life and increased prevalence of long-term health complications. Diabetes affects almost 3.7 million people in the UK [1], a figure that continues to rise [2] despite efforts to promote healthier lifestyle changes and developments in pharmacological interventions. It is predicted that by 2035, diabetes will account for 17% of the National Health Service (NHS) expenditure [3].

A substantial body of evidence demonstrates the benefits of structured self-management education (SSME) in T2DM [4, 5]. Here, the term SSME is used to encompass any education programme meeting the recommended National Institute for Health and Care Excellence (NICE) criteria [6], regardless of the mode of delivery. SSME has been shown to be both cost-effective [4] and result in improved biomedical and psychological outcomes [5, 7, 8]. Improvements have been seen in HbA1c, lipids, weight and blood pressure, as well as in depression and quality of life [5, 7, 8]. It is therefore unsurprising that NICE recommends SSME to be made available to patients “at and around the time of diagnosis, with annual reinforcement and review” [9]. However, despite the demonstrated benefits of SSME and it being made a national priority by NICE, rates of uptake to SSME for those with T2DM have remained low. Latest figures show that although the number of patients with T2DM-offered SSME has increased substantially in the last 5 years, and as of 2015, this figure was in excess of 90%, in the same year, less than 10% of those diagnosed with T2DM were recorded as having attended [10]. A systematic review of the reasons why patients referred to diabetes education programmes choose not to attend found two broad categories of non-attenders [11]. The authors found the first category to comprise patients who were unable to attend due to social or logistical reasons, whilst the second category comprised those patients who chose not to attend or perceived no benefit in doing so [11]. The review concluded that the referrers had a responsibility to ensure that “those who are referred are appropriate, ready and fully informed”, whilst the courses that they are referred to should be ‘accessible in time and place wherever possible’ [11].

There is now a burgeoning literature on the ways in which health care interventions are implemented in a variety of organisational settings, yet authors have highlighted the absence of studies which focus on the longer-term sustainability of such interventions [12–14]. The question of how improvements in health care are retained and become embedded or ‘routinised’ in everyday practice remains poorly understood, and is as much a matter of networks of influence and knowledge of priorities and incentive frameworks than as of clinical or cost-effectiveness in themselves [13]. It is important, therefore, to explore how to ensure that SSME can become part of routine care in the new organisational structures of the NHS in ways that are feasible for all stakeholders. This requires a better understanding of the contextual factors and processes that encourage the adoption of structured education interventions and what the barriers might be to longer term sustainable change and how to overcome these barriers.

To address this need, an intervention to increase uptake to SSME was developed. In accordance with guidance from the UK Medical Research Council [15], multiple phases of work were undertaken using a multiple-method approach, informed by the Normalisation Process Theory (NPT) [16]. Three stages of work were undertaken over a 12-month period. The process was iterative, with each stage informing subsequent stages. Stage one [an evidence synthesis comprising a review of 23 published articles and a secondary analysis of five qualitative data sets (*n* = 74 interviews) concerning referral to and uptake of SSME for T2DM] intended to understand how and why stakeholders engage with and participate in SSME for T2DM and identify barriers and enablers to SSME referral and uptake. Next, a guided discussion (Stage two) was facilitated among the research team, primary care stakeholders and providers of SSME to understand which characteristics of an SSME programme are prioritised by stakeholders, as well as the resources required to address barriers and deliver solutions. Finally, in Stage three, a purposive sample of 16 individuals with a professional interest in SSME, management of chronic health conditions, or the implementation and adoption of interventions in primary care were recruited to participate in a consultation to rank and select the key functions of an intervention to increase referral to and engagement with SSME for T2DM.

## The Embedding Package

A theory-and-evidence-based intervention (the Embedding Package) was developed to overcome the previously identified barriers to the uptake of SSME. The Embedding Package incorporates four key components: (1) a clear marketing strategy for SSME, (2) a user friendly and effective referral pathway, (3) new/amended professional roles, including local clinical champions and an ‘Embedder’, and (4) a toolkit of resources, complemented by a website. In the operation of the intervention, the Embedder guides practices and SSME providers and educators through the implementation process. Tailoring of the intervention to the local context is achieved through a series of meetings between stakeholders and the Embedder.

The appointment of an ‘Embedder’ was part of the new/amended professional roles. The role of the Embedder is to liaise between all relevant stakeholders to promote SSME and use of the resources. A local champion, identified in each CCG, will promote SSME and the Embedding Package at both practice and CCG level.

Promotional resources targeting different stakeholders will include (a) patient-facing resources such as promotional posters, SSME invitation letters and self-referral forms; (b) resources for health care professionals including document templates, guidance on recruiting staff, referring patients and increasing staff engagement; and (c) resources for education providers and commissioners that will consist of electronic administration and referral systems, sample referral pathways and evidence summaries. In addition, the toolkit will also include guidance on communication strategies, auditing, conducting local needs assessments, as well as details of how to ensure patient accessibility and tailoring of SSME courses and sessions.

The overall aim of the study presented here was to test feasibility of conducting a large-scale evaluation of the Embedding Package in primary care using a mixed methods approach. This was achieved through the following objectives:

Quantitative:

1. To assess the feasibility of two recruitment and consent approaches
2. To assess the feasibility of extracting demographic and biomedical data and information on SSME referral and attendance from primary care medical records with sufficient accuracy and completeness for use in an RCT, particularly HbA1c, the primary outcome for the RCT.
3. To assess patient willingness to provide consent for accessing and extracting identifiable data from their medical records
4. To assess the willingness of patients to provide consent and complete a questionnaire asking for demographic data and information on the diagnosis and management of their diabetes, their history of being invited to or attending SSME and their preferences around the method of delivery of SSME
5. To assess the feasibility of capturing cost data for embedding activities at participating practices and clinical commissioning groups (CCGs)

Qualitative:

1. To assess the feasibility of using ethnographic methods in a range of primary care settings
2. To identify and collect context-specific data on the processes of implementation, sustainability of change, and the fit of the Embedding Package with routine practice
3. To evaluate the application of the Normalisation Process Theory (NPT) as a way of analysing data and understanding factors contributing to the embedding of SSME
4. To explore methods for providing formative feedback to aid in the refinement of the Embedding Package and development of RCT study procedures

## Methods

### Study design

**Fig. 1:**
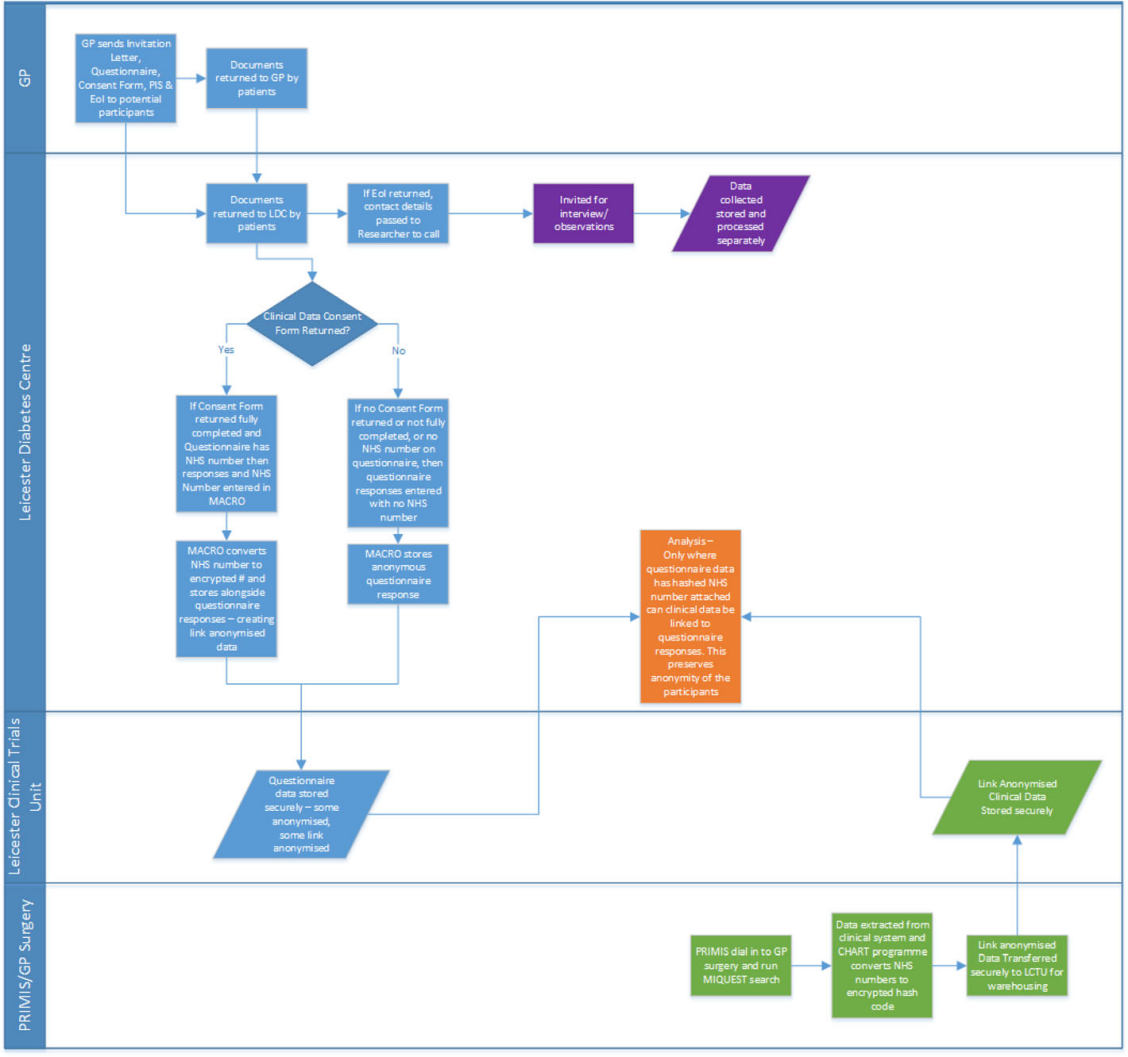
Flow diagram of the quantitative data collection process

The feasibility study involved collecting data from six general practices and two SSME providers within two CCGs in the East Midlands, UK, between May and August 2017. Figure 1 provides an overview of the quantitative data collection process.

Quantitative demographic and biomedical data and information relating to SSME referral and attendance and diabetes diagnosis and management were gathered from primary care electronic medical records (extracted data), SSME providers (health economic data) and questionnaires completed by patients with T2DM (self-reported data). An integrated concurrent ethnographic study was undertaken, incorporating observations, interviews and document analysis. The use of qualitative methods enabled an understanding of the implementation and adoption of the Embedding Package within the social, environmental and organisational context and facilitated theoretical evaluation of intervention components [17]. In addition, data relating to the cost of implementing the intervention were obtained from the practice managers and an appropriate member of staff in the CCG, using a questionnaire and interview.

### Sample

Three groups of participants were included in this research: general practice staff, patients and staff in organisations providing SSME. As this was a feasibility study, there was no formal sample size calculation. The number of practices was based on a balance between practicalities and having sufficient data to assess its quality and to give adequate variation in urban and rural practices. It was anticipated that six practices would provide data extracted from around 4200 patients. This was based on an average diabetes register size of 700, informed by figures from a Quality and Outcomes Framework report [18]. All eligible patients in these included practices were invited to participate in the questionnaire. It was assumed that 30% of the patients on the diabetes register would attend their surgery during the recruitment window and be given study information by a health care professional. It was estimated that there would be a 20% questionnaire return rate for this group, so this would collect data from 250 patients. All remaining eligible patients with T2DM would receive a postal questionnaire; it was estimated that 10% of these would be returned. This would result in a total of ~ 540 patient participants returning a questionnaire with implied consent. This sample would then be large enough that a sufficient number of patients would have returned a completed questionnaire and agreed to data linkage, to allow assessment of the agreement between the two data sources.

For the integrated ethnographic component, a purposive approach to sampling aimed to achieve a range of staff and patients, in terms of roles demography and experience, within the size and time limits of the wider study. The aim was to recruit between 12 and 15 patient participants and conduct between six and ten interviews with participants representing practices and providers. Participant recruitment continued until data saturation was reached.

As we did not intend to generate inference from our qualitative data, there was no optimum number of participants. Rather, we invited participation from individuals who could provide the most productive information, based on a framework of variables [maximum variation (patients) and key informants (and where appropriate deviant cases)], our practical knowledge of the research area, and existing literature on primary care implementation research. In addition to relying on saturation of data [19], we were also guided by principles of adequacy, appropriateness and analytical redundancy—in that one or many more interviews would not have made additional contributions or provide further insight [20].

### Eligibility criteria

General practices using either EMIS Web or TPP SystemOne electronic health record systems and located within the two participating CCGs were invited to participate in the study. Practices had to be referring patients with T2DM to an SSME programme and be willing to sign a data sharing and remote data collection agreement with PRIMIS (a third party company providing the data extraction service for the study). This allowed the collection of anonymised patient data, and where consent had been provided by patients’ identifiable data. Since this study did not aim to assess the effectiveness of the intervention, a comparator was not required, and all general practices (and patients with T2DM within them) received the intervention.

Patients from participating practices were eligible to have their anonymised data extracted if they were aged 18 years or over and coded in their electronic medical record as diagnosed with T2DM. Patients with a record of a terminal illness and life expectancy of < 12 months, housebound or in residential care, or with a dissent code to data sharing as part of a research study, were excluded. If the above criteria were met, and the patient was able to provide informed consent and able to speak, understand, and read English to a sufficient level to participate in the study, they were invited to complete a questionnaire and an additional consent form, allowing their data extracted by PRIMIS to be linked to their questionnaire response.

Health care professionals were eligible for inclusion if they were employed by a participating practice or an organisation referring to, providing, or commissioning SSME, and willing and able to provide informed written or verbal consent.

### Patient recruitment and consent

#### Quantitative study

Participants were recruited in two ways. Firstly, through the use of a prompt installed on the electronic health record system, which notified the clinician during a consultation of a patient’s potential eligibility for inclusion in the study. This encouraged the clinician to make the patient aware of the study and provide them with a participant information pack and a copy of the data collection questionnaire for self-completion. The second method involved postal invitations to participate. All potentially eligible patients who had not been invited in person received an information pack and self-completion questionnaire. Informed consent was implied by the return of a completed questionnaire directly to the study team using a pre-paid, self-addressed envelope.

The questionnaire could be completed anonymously as questions asking for sex, year of birth and ethnicity were optional. For patients opting to share and link their health record data to their questionnaire responses, additional written informed consent was sought and obtained.

#### Concurrent qualitative studies

Informed written consent was obtained from all participants undertaking interviews. Informed verbal consent was sought and obtained prior to and during all observations. No incentives or disbursements were provided to participants.

### Data collection

#### Quantitative data collection

Pseudonymised patient data were extracted from primary care medical records for eligible patients by a third party (PRIMIS). Data were extracted from each practice at a single time point in September or October 2017. Data extraction procedures were based on those used by a similar study [21] and extracted data recorded over the previous 12 months. Information was extracted on age, sex, ethnicity, date of T2DM diagnosis, HbA1c (mmol/mol), body mass index (BMI), weight, lipids (total and HDL), blood pressure (SBP and DBP), medications, atrial fibrillation, smoking status, hospital admissions and cardiovascular risk (QRisk2) score. The last value recorded within the last 12 months was extracted for all biomedical/anthropometric data. Any Read coded data relating to offering, referring, uptake or did not attend (DNA) of SSME at any time since diagnosis, with dates, whether the patient had been approached to take part in the questionnaire, and whether they declined participation in the questionnaire study was also collected.

The questionnaire gathered data on registered practice, diagnosis and management of diabetes (time since T2DM diagnosis, HbA1c and diabetes medication), history of being invited to SSME and of attendance at SSME, including delivery method. The questionnaire also asked for patient preferences around the delivery of SSME, i.e. web-based or face-to-face. Basic demographic information was also sought on year of birth, sex and ethnicity; however, responses to these items were optional.

#### Qualitative data collection

Traditional ethnographic methods were used to collect data (key-informant and semi-structured interviews, naturalistic observation and collection of relevant documents and publically available information). The aim was to identify methodological barriers and facilitators, develop feedback processes between the qualitative and wider study team, provide insight into quantitative outcomes and contribute to the refinement of intervention content, processes and definitive trial procedures—doing so in a manner that was both rigorous and acceptable to primary care stakeholders and patients.

To gain insight into clinical and organisational culture and context, we collected relevant information and documentation as appropriate. Practice websites, Care Quality Commission reports and local authority public health Joint Strategic Needs Assessment reports were reviewed to provide contextual information in relation to practice size, services and local area demographic. Diabetes- and diabetes education-related patient information materials were also collated. Meeting notes and task plans developed between the Embedder and practices or Educators were also reviewed and contributed to the final analysis. Action planning and action planning review documents were analysed to assess the development of embedding activity, the delegation of roles and responsibilities and outputs.

NPT informed data collection instruments [16]. Guides were designed to facilitate observations and interview discussions. The observational guide aided our understanding of the intervention implementation process, and a schedule of topics for discussion (Additional File 1) explored the variety of experiences of each participant group in relation to intervention implementation, outcomes and barriers and enablers to adopting the toolkit.

Observations were undertaken totalling approximately 25 h of practice and provider meetings and activities, taking field notes in situ*,* which were then transcribed to provide a full and rich account. Ethnography team debrief meetings were held to reflect on the observations and interviews and identify salient points for future exploration in interviews. Tensions, inconsistencies, or ambiguities in the data were also identified.

Individual and group interviews with stakeholders (patients, practice staff and SSME providers) were held and the Embedder was interviewed at three-month intervals to explore perceptions of the implementation process, including delivery and use of the toolkit and outcomes. All interviews were audio-recorded with participant consent and transcribed verbatim. Demographic data on participant characteristics were collected using a brief questionnaire.

#### Cost data collection

A questionnaire and face-to-face interview were used to obtain data on the cost of all the embedding activity from the practice manager and an appropriate member of staff in the CCG. A simple tick box tracker covered the type of embedding activity implemented, the duration over which it was applied and whether it was still ongoing. This tracker was completed by the practice and the ‘Embedder’. This provided a census of what activities were attempted as well as providing a measure of resource use in terms of time spent by the Embedder and staff from each provider and practice, against which unit costs could be applied. The unit cost was generated by the practice manager interviews which asked for details of staff time, consumables and other costs that have been devoted to each individual activity over the duration that the activity was undertaken. The resource use data for developing the web-based toolkit (a key component of the Embedding Package) were obtained from the designer.

### Analysis

#### Statistical analysis

##### Quantitative data

The data were analysed using descriptive summary statistics to assess data completeness and accuracy using mean, standard deviation (SD) and range for continuous, normally distributed variables, median, interquartile range and range for continuous, non-normally distributed variables and number (percentage) for categorical variables. Extracted primary care data were summarised overall, by CCG and by practice to identify whether there were CCG or practice-level variations in recording. Data from the questionnaire were summarised using the same descriptive statistics. For the participants who had consented to data linkage, their questionnaire responses were linked to their practice data using the pseudonymised NHS number. Where the same categorical measure was available for both the questionnaire and primary care data, these were cross-tabulated and the percentage agreement, calculated. In this comparison, missing values were ignored. For continuous variables, mean difference and limits of agreement were calculated. All analyses were performed in Stata 14.1.

For the practice activity tracker, a cross-tabulation of activity by practice was undertaken, together with the calculation of the mean duration of each activity across practices. For the practice manager interviews, central estimates of resource use for each activity are reported, together with upper and lower bounds where there was uncertainty relating to the best estimate. Estimates of resource use for each activity in each practice were multiplied by relevant unit costs and used to produce an average cost per activity over time; this was then divided by the number of patients with T2DM at the practice to get a per-patient cost. Resource use data were valued by applying unit costs using the University of Kent’s Personal Social Services Research Unit (PSSRU) unit costs 2016 and other sources where relevant (Additional File 2) [22, 23].

#### Qualitative data analysis

A thematic framework approach to the analysis of the complete data corpus was adopted, to enable comparison within and between cases [24]. Management of data were undertaken in NVivo. Analysis followed the procedure described by Gale and colleagues [24]:

I. Familiarisation with the transcript data: This stage involved the reading, re-reading and open coding of interview and debrief transcripts, supplemented by the additional documentation to become familiar with the data. Additionally, notes were made on the transcripts to reflect any first impressions or thoughts.
II. Coding: Open (inductive) coding was conducted independently by two researchers and aimed to classify all of the data; preliminary themes were then organised and charted to identify accounts that differed from the rest, or that which challenged or reconciled anomalies in accounts.
III. Development of an analytical framework: After coding and organising the data, initial themes were organised by the four domains of NPT. Different approaches to grouping and defining different stakeholder data were considered.
IV. Application of the analytical framework: Using the descriptors of the NPT domains [16] as guidance, narrative data summaries were produced that incorporated narrative and documentary data. After making several attempts to logically organise data, data were categorised and presented according to stakeholder category (patient, practice, provider and the Embedder).
V. Data interpretation: Throughout the analysis process, the entire ethnography team met in a series of analysis meetings to review findings and procedures. These discussions formed the basis of an agreed coding framework and where there was uncertainty or disagreements concerning coding, these were resolved through further discussion and consensus between the research team.

## Results

### Assessment of the feasibility of two recruitment and consent approaches

Two CCGs were approached; four expressions of interest were received from one CCG and six from the other. Six of these practices were then selected to participate in the study and agreed to the extraction of their primary care data. Eligible patients were then approached to participate in the questionnaire, either by GP prompt or postal invite. After the removal of duplicates, 423 unique questionnaires (response rate 14.7%) were received across the six practices (66 patients per practice on average; range = 8 to 102). Twenty eight patients did not have a practice recorded (i.e. they returned the questionnaire anonymously). More participants were recruited via the postal invite (85%) than the GP prompt (15%).

Of those returning a questionnaire, 384 (90.8%) consented to have their questionnaire responses and primary care data linked. Of these, 20 (5.2%) could not be linked because they did not provide their NHS number and eight (2.1%) because they did not have their primary care record extracted (three of these were recruited via their GP and five via postal invitation).

### Assessment of the feasibility of extracting demographic and biomedical data, and information on SSME referral and attendance from primary care medical records

Primary care data were extracted for all six practices and for 2877 patients [mean = 479.5 patients per practice (SD = 262.3); median = 459 (range = 118 to 824)]. All patients had a T2DM code and were aged 18 years or older as required, but it was not possible to verify the other eligibility criteria from the extracted data. The extracted data are summarised overall and by CCG in Tables 1 and 2 and by practice in Additional File 3.

**Table 1:** Summary of extracted continuous primary care data overall and by CCG

	All (*N* = 2877)			CCG 1 (*N* = 1036)		CCG 2 (*N* = 1841)	
Variable	*N* (%) missing	Mean (SD)	Median (range)	*N* (%) missing	Mean (SD)	*N* (%) missing	Mean (SD)
Age, years	0 (0)	66.0 (13.3)	67 (23, 101)	0 (0)	68.6 (12.5)	0 (0)	64.6 (13.5)
HbA1c, mmol/mol	189 (7)	56.1 (15.5)	52 (29, 179)	22 (2)	55.0 (13.4)	167 (9)	56.8 (16.6)
HbA1c, %	189 (7)	7.3 (1.4)	6.9 (4.8, 18.5)	22 (2)	7.2 (1.2)	167 (9)	7.3 (1.5)
Weight, kg	622 (22)	88.1 (21.9)	85.7 (1.5*, 215.0)	89 (9)	85.9 (20.4)	533 (29)	89.7 (22.8)
Total cholesterol, mmol/L	349 (12)	4.3 (1.1)	4.2 (1.9, 13.4)	67 (6)	4.3 (1.1)	282 (15)	4.3 (1.1)
HDL cholesterol, mmol/L	487 (17)	1.3 (0.4)	1.2 (0.2, 4.4)	73 (7)	1.3 (0.3)	414 (22)	1.3 (0.4)
Systolic blood pressure, mmHg	204 (7)	132.5 (13.7)	132 (86, 240)	41 (4)	133.7 (13.8)	163 (9)	131.8 (13.6)
Diastolic blood pressure, mmHg	204 (7)	75.1 (9.5)	76 (45, 134)	41 (4)	75.2 (9.6)	163 (9)	75.1 (9.4)
QRisk score, %	1468 (51)	23.1 (15.4)	19.9 (0, 98.7)	669 (65)	25.4 (15.4)	799 (43)	22.3 (15.3)

*HDL* high-density lipoproteins, *CCG* clinical commissioning group, *SD* standard deviation

*Removing outliers minimum weight was 39.7 kg

**Table 2:** Summary of extracted categorical primary care data overall and by CCG

Variable	*N* (%)		
	All (*N* = 2877)	CCG 1 (*N* = 1036)	CCG 2 (*N* = 1841)
Sex			
Male	1588 (55.2)	575 (55.5)	1013 (55.0)
Female	1289 (44.8)	461 (44.5)	828 (45.0)
Ethnicity			
White European	1948 (67.7)	618 (59.7)	1330 (72.2)
South Asian	219 (7.6)	56 (5.4)	163 (8.9)
Black	57 (2.0)	5 (0.5)	52 (2.8)
Other	32 (1.1)	12 (1.2)	20 (1.1)
Not otherwise stated	121 (4.2)	102 (9.9)	19 (1.0)
Missing	500 (17.4)	243 (23.5)	257 (14.0)
Smoking status			
Never smoker	1425 (49.5)	508 (49.0)	917 (49.8)
Ex-smoker	1102 (38.3)	435 (42.0)	667 (36.2)
Current smoker	350 (12.2)	93 (9.0)	257 (14.0)
HbA1c, mmol/mol (%)			
≤ 53 (≤ 7%)	1465 (50.9)	585 (56.5)	880 (47.8)
54–58 (7.1–7.5%)	385 (13.4)	148 (14.3)	237 (12.9)
59–64 (7.6–8.0%)	279 (9.7)	100 (9.7)	179 (9.7)
65–69 (8.1–8.5%)	152 (5.3)	58 (5.6)	94 (5.1)
70–86 (8.6–10.0%)	266 (9.3)	83 (8.0)	183 (9.9)
≥ 87 (≥ 10.1%)	141 (4.9)	40 (3.9)	101 (5.5)
Missing	189 (6.6)	22 (2.1)	167 (9.1)
Diabetes medication in the last 12 months			
None recorded	639 (22.2)	215 (20.8)	424 (23.0)
DPP-IV	101 (3.5)	22 (2.1)	79 (4.3)
GLP-I	20 (0.7)	10 (1.0)	10 (0.5)
Insulin	263 (9.1)	80 (7.7)	183 (9.9)
Metformin	1425 (49.5)	559 (54.0)	866 (47.0)
SGLT-2	21 (0.7)	5 (0.5)	16 (0.9)
Sulphonylurea	346 (12.0)	136 (13.1)	210 (11.4)
Multiple diabetes medications	38 (1.3)	0 (0.0)	38 (2.1)
Other	24 (0.8)	9 (0.9)	15 (0.8)
Previous atrial fibrillation diagnosis			
Yes	246 (8.6)	106 (10.2)	140 (7.6)
No	2631 (91.5)	930 (89.8)	1701 (92.4)
SSME referral			
No record	1809 (62.9)	546 (52.7)	1263 (68.6)
DESMOND	586 (20.4)	106 (10.2)	480 (26.1)
X-PERT	2 (0.1)	0 (0.0)	2 (0.1)
DAFNE	18 (0.6)	2 (0.2)	16 (0.9)
Generic SSME code	462 (16.1)	382 (36.9)	80 (4.4)
Date of SSME referral			
≤ 1 year	270 (9.4)	138 (13.3)	132 (7.2)
> 1 year	798 (27.7)	352 (34.0)	446 (24.2)
Missing	1809 (62.9)	546 (52.7)	1263 (68.6)
SSME attendance			
Not referred	1317 (45.8)	311 (30.0)	1006 (54.6)
Attendance not recorded	403 (14.0)	266 (25.7)	137 (7.4)
Did not attend	744 (25.9)	395 (38.1)	349 (19.0)
Attended	413 (14.4)	64 (6.2)	349 (19.0)
Date of SSME attendance			
≤ 1 year	121 (4.2)	12 (1.2)	108 (5.9)
> 1 year	291 (10.1)	52 (5.0)	239 (13.0)
Missing	2465 (85.7)	972 (93.8)	1493 (81.1)

*CCG* clinical commissioning goup, *SSME* structured self-management education, *DPP-IV* dipeptidyl peptidase 4, *GLP-I* glucagon-like peptide-1, *SGLT-2* sodium-glucose transport protein 2

Regarding SSME referral data, 63% of patients had no record of ever being referred to an SSME programme, 20% had been referred to DESMOND, and 16% did not have a specific type of course entered (i.e. the generic SSME Read code had been used which is the recommended code). The majority of patients’ last referral had been over a year previously. There were 18 spurious referrals to SSME where the patients were referred to DAFNE, which is a course for patients with type 1 diabetes, not T2DM.

Regarding SSME attendance data, 46% of patients were not recorded as being referred to SSME. This is lower than the 63% estimate based on the referral data. The discrepancy arises because where patients were recorded as attending or declining SSME, it was assumed that they had been referred to SSME (*n* = 492 patients). Only 14% of patients were recorded as having attended SSME, 26% did not attend, and 14% were referred but had no GP record as to whether or not they subsequently attended.

CCG 1 had more referrals recorded than CCG 2 (47% vs 31%, respectively) but lower attendance recorded (absolute values: 6% vs 19%, respectively, indicating that 13% of referrals in CCG1 resulted in attendance compared with 60% in CCG 2). This difference may be as a result of differing “quality” of the offer of SSME.

#### Accuracy of variables

Regarding accuracy of these variables, mean and SD estimates were similar for all continuous variables when summarised overall, by CCG and by practice, suggesting that there were no consistent reporting errors in any of the practices or CCGs. The only exception to this was mean QRisk score which ranged from 17.4% to 28.3% between the six practices. On the range checks, the only spurious values identified were for weight which had two likely outliers (< 0.001% of values) of 1.5 kg and 8.8 kg.

#### Missing data

Regarding completeness of demographic and biomedical data, age, sex and smoking status had no missing values. HbA1c and blood pressure had less than 10% missing data, but there were more missing data for total (12%) and HDL (17%) cholesterol, ethnicity (17%), weight (22%) and QRisk score (51%). BMI and hospital admissions were missing for all patients.

There were some fairly large differences in the percentages of missing data between CCGs. For example, weight was missing for 9% of patients in CCG 1 and for 29% in CCG 2. Similarly, cholesterol was missing for a higher percentage of patients in CCG 2 than in CCG 1, whereas the higher percentage of missing QRisk scores was in CCG 1. Examining the summary by practice suggests that these differences are driven by one or two practices with particularly high percentages of missing values. For example, Practice 3 had only 24% missing QRisk scores, whereas Practice 2 had 71% missing QRisk scores.

### Assessment of patient willingness to provide consent and complete the questionnaire

The questionnaire had a 14.7% response rate across the six practices, receiving 423 unique responses, which are summarised in Table 3. An average of 66 patients per practice (range 8 to 102) completed the questionnaire.

**Table 3:** Summary of patient questionnaire data (*n* = 423)

**Characteristic**	***N*missing values**	**Mean (SD)**
Age, years	6	68.3 (11.1)
	**Categories**	**N(%)**
Recruitment source	General practitioner	64 (15.1)
	Postal invite	359 (84.9)
Sex	Male	243 (57.5)
	Female	179 (42.3)
	Missing	1 (0.2)
Ethnicity	White	385 (91.0)
	South Asian	21 (5.0)
	Black	8 (1.9)
	Other	6 (1.4)
	Missing	3 (0.7)
Time since type 2 diabetes diagnosis	< 12 months	19 (4.5)
	1 to 3 years	29 (6.9)
	4 to 10 years	146 (34.5)
	> 10 years	220 (52.0)
	Missing	9 (2.1)
HbA1c measured in the last 12 months	Yes	392 (92.7)
	No	13 (3.1)
	Do not know	16 (3.8)
	Missing	2 (0.5)
HbA1c result, mmol/mol	≤ 53 (≤ 7%)	136 (32.2)
	54–69 (7.1–8.5%)	82 (19.4)
	70–86 (8.6–10.0%)	19 (4.5)
	≥ 87 (≥ 10.1%)	8 (1.9)
	Missing	178 (42.1)
Diabetes medication	None (diet and lifestyle)	97 (22.9)
	DPP-IV	7 (1.7)
	GLP-I	1 (0.2)
	Insulin	24 (5.7)
	Metformin	145 (34.3)
	SGLT-2	1 (0.2)
	Sulphonylurea	10 (2.4)
	Multiple diabetes medications	121 (28.6)
	Other	0 (0.0)
	Missing	17 (4.0)
Ever referred to SSME	No	158 (37.4)
	Yes	241 (57.0)
	Do not know	14 (3.3)
	Missing	10 (2.4)
Date of SSME referral	≤ 1 year	55 (13.0)
	> 1 year	166 (39.2)
	Do not know	5 (1.2)
	Missing	197 (46.6)
Ever attended group SSME	No	211 (49.9)
	Yes	190 (44.9)
	Missing	22 (5.2)
Type of SSME attended	N/A	233 (55.1)
	Unknown	20 (4.7)
	DESMOND	72 (17.0)
	Juggle	53 (12.5)
	Tonic	1 (0.2)
	Other	21 (5.0)
	Missing	22 (5.2)
Date of SSME attendance	≤ 1 year	29 (6.9)
	> 1 year	116 (27.4)
	Missing	278 (65.7)
Re-invited if did not attend	No	128 (30.3)
	Yes	11 (2.6)
	Missing	284 (67.1)
Reason for not attending SSME	Lack of information	2 (0.5)
	Lack of perceived benefit	16 (3.8)
	Unsuitable time	5 (1.2)
	Suitable transport unavailable	3 (0.7)
	Other^a^	33 (7.8)
	Rather not say	1 (0.2)
	Missing	363 (85.8)
Ever attended one-to-one SSME	No	372 (87.9)
	Yes	21 (5.0)
	Do not know	15 (3.6)
	Missing	15 (3.6)
Ever attended online SSME	No	403 (95.3)
	Yes	8 (1.9)
	Do not know	2 (0.5)
	Missing	10 (2.4)
Would you attend SSME if invited?	No	99 (23.4)
	Yes	299 (70.7)
	Missing	25 (5.9)
Reason for not wanting to attend if invited	Lack of information	1 (0.2)
	Lack of perceived benefit	24 (5.7)
	Unsuitable time	3 (0.7)
	Suitable transport unavailable	9 (2.1)
	Other^b^	44 (10.4)
	Rather not say	1 (0.2)
	Missing	341 (80.6)
Preferred format	Group	103 (24.4)
	One-to-one	122 (28.8)
	Online	64 (15.1)
	Group/one-to-one	39 (9.2)
	Group/online	9 (2.1)
	One-to-one/online	7 (1.7)
	Any	12 (2.8)
	Missing	67 (15.8)

*SD* standard deviation, *SSME* structured self-management education

^a^These reasons are listed in Additional File 4

^b^These reasons are listed in Additional File 5

#### Demographics

The questionnaire participants were representative of the eligible population (i.e. those for whom primary care data were extracted) in terms of mean age [68 (SD = 11.1) and 66 (SD = 13.3) years, respectively) and sex distribution (58% and 55% male, respectively). White participants were however over-represented in the questionnaire population compared to those for whom primary care data were extracted (91% and 68%, respectively). Over half (52%) of the questionnaire participants had long-standing T2DM (> 10 years). Time since diagnosis was not available in the primary care data for comparison.

#### Management of diabetes

For HbA1c, the percentage reporting that they had had it measured in the last 12 months (93%) was the same as in the primary care data (93%). Self-reported HbA1c was missing for 42% of participants. However, when missing data were excluded, the percentages of patients with HbA1c ≤ 53 mmol/mol were very similar in the self-report (56%) and primary care (55%) data, suggesting that self-reported HbA1c data were incomplete but accurate.

Another notable difference between the primary care and self-report data is that 29% self-reported being prescribed with multiple diabetes medications, whereas only 2% were recorded as such in the primary care data. Metformin was the most common medication (47% primary care and 34% self-reported). A similar proportion reported not being on any medication in the primary care and self-reported data (23% and 22.9% respectively).

#### Referrals and attendance at SSME

Regarding SSME, 37% of patients reported never having been referred compared with 63% in the extracted primary care data. The percentage who self-reported attendance at group SSME (45%) was much higher than that recorded in the extracted primary care data (14%).

In relation to the question, ‘Have you ever attended a one-to-one session with an educator to teach you about your diabetes (this would be outside of the normal care you receive at your GP practice)?’, 17 of the 21 participants who responded positively reported that this had happened as part of their normal care at their practice, and so had incorrectly answered the question. Among the eight participants who reported attending an online SSME programme, four completed a Diabetes UK online programme.

The majority of the participants (70%) reported that they would attend SSME if invited. The preferred formats were one-to-one (29%) or group (24%). Fewer participants preferred a format including an online component (online, group/online or one-to-one/online; 19%) than face-to-face only formats (group, one-to-one or group/one-to-one; 62%).

### Assessment of patient willingness to provide consent for accessing and extracting identifiable data from their medical records

In total, 356 participants were included in the linked analyses, which are summarised in Table 4.

**Table 4:** Agreement between primary care and patient questionnaire data (*n* = 356)

**Characteristic**	***N***	**Mean difference**	**95% Limits of agreement**	
Age, years	352	0.32	− 2.17, 2.81	
HbA1c, mmol/mol	40	− 0.66	− 11.33, 10.00	
**Characteristic**	**Categories**	***N*(%) agree**	***N*(%) disagree**	**% Agreement**
Sex	Male	212 (99.5)	1 (0.5)	
	Female	142 (100.0)	0 (0.0)	99.7
Ethnicity	White	256 (93.8)	17 (6.2)	
	South Asian	13 (81.3)	3 (18.7)	
	Black	5 (83.3)	1 (16.7)	
	Other	1 (50.0)	1 (50.0)	92.6
HbA1c measured in the last 12 months	Yes	323 (97.9)	7 (2.1)	
	No	9 (81.8)	2 (18.2)	94.7
HbA1c result, mmol/mol	≤ 53 (≤ 7%)	108 (93.1)	8 (6.9)	
	54–69 (7.1–8.5%)	43 (61.4)	27 (38.6)	
	70–86 (8.6–10.0%)	5 (33.3)	10 (66.7)	
	≥ 87 (≥ 10.1%)	2 (28.6)	5 (71.4)	76.0
Diabetes management	Diet and lifestyle	71 (88.9)	9 (11.3)	
	DPP-IV	5 (71.4)	2 (28.6)	
	GLP-I	1 (100.0)	0 (0.0)	
	Insulin	15 (75.0)	5 (25.0)	
	Metformin	118 (96.7)	4 (3.3)	
	SGLT-2	1 (100.0)	0 (0.0)	
	Sulphonylurea	8 (100.0)	0 (0.0)	
	Multiple diabetes medications	3 (2.9)	101 (97.1)	
	Other	0 (0.0)	0 (0.0)	55.9
SSME referral	No	101 (76.5)	31 (23.5)	
	Yes	111 (54.2)	94 (45.9)	59.6
Date of SSME referral	≤ 1 year	26 (76.5)	8 (23.5)	
	> 1 year	67 (91.8)	6 (8.2)	86.9
SSME attendance	No	169 (96.6)	6 (3.4)	
	Yes	78 (46.4)	90 (54.6)	72.0
Date of SSME attendance	≤ 1 year	13 (100.0)	0 (0.0)	
	> 1 year	37 (82.2)	8 (17.8)	86.2

*SSME* structured self-management education

High levels of agreement were demonstrated for the demographic variables: age (mean difference = 0.3 years), sex (~ 100.0% agreement) and ethnicity (93% agreement). The large discrepancy between multiple diabetes medications described above is also present in the linked data with 97% of those who self-reported taking multiple diabetes medications having only one medication listed in their extracted data over the previous 12 months.

#### Management of diabetes

Most participants were aware whether they had had HbA1c measured in the last 12 months (98% agreement), but self-reporting of HbA1c values was fairly poor. For example, the mean difference was small (− 0.6 mmol/mol), but the 95% limits of agreement were large ranging from − 13.1 mmol/mol (− 3.4%) to 12.0 mmol/mol (3.2%). There seemed to be a particular problem with participants reporting high HbA1c values (≥ 70 mmol/mol; 8.6%) that were not reflected in their primary care record. This may be due to participants confusing glucose measurements with measurements of HbA1c.

#### Referrals and attendance at SSME

For SSME referrals, agreement was moderate (60%). Of those who self-reported that they had not been referred, 77% also had no record of referral in their primary care data. However, of those who self-reported that they had been referred, only 54% had a record of this in their primary care data suggesting that not all of the discrepancy between the two sources is due to the representativeness of the questionnaire participants. For SSME attendance, there was high agreement between the two sources when the participant self-reported that they had not attended (97%). However, when the participant reported that they had attended SSME, there was only a primary care record of this for 46% of the participants. Where both sources had a record of referral and attendance, there was high agreement as to when this had occurred (87% and 86% respectively).

### Feasibility of capturing cost data for Embedding activities at practices and CCGs

#### Initiative tracker and proforma completion

Resource use and cost data were collected for all participating practices, CCGs and SSME providers based on the Embedder’s tracker. Each of the pre-specified intervention components (marketing, referral and data collection, champion, administrator, and web-based toolkit) was attempted by at least one practice over the study follow-up. However, none of the CCG designates and practice managers completed the intervention tracker or proforma.

#### Interviews

One GP practice agreed to take part in the face-to-face interview with the Health Economist to discuss resource use and costs incurred to allow the Embedding intervention to take place. The interview was undertaken with the Champion (a senior health care professional) and practice administrator who were both involved in implementing the feasibility study at practice level. Since no intervention activity tracker or proforma was completed in advance of the meeting, the interview was based on the information from the Embedder’s tracker record for the practice (e.g. staff time spent in providing each activity). The interview provided the information required for estimating unit costs for valuation of resource use at the practice level (e.g. diabetes awareness event, providing resources in different languages to patients, photocopying).

#### Cost estimation

For the six practices, the median period over which resource use data was recorded was 162.5 days (range: 144 to 191). For CCG and SSME providers, the median follow-up was 152 days (range: 150 to 154). The mean total cost per practice over the study period was able to be estimated as £3363 (SD = 2140), with an average cost per patient of £8.94 across all practices. However, if the cost for developing the Embedding Package toolkit is removed from this calculation, the mean total cost per practice is reduced. The Embedding intervention will be tested across 66 general practices in the full RCT (sample size informed by the average practice size for this feasibility study), and if shown to be effective, the toolkit will be provided across the whole NHS [25]. Therefore, it is felt the development cost per practice for this feasibility study provides an unrealistic real-world cost per practice.

The breakdown of costs for each individual activity by CCG/SSME provider and practice and the mean cost per patient (weighted by the number of patients with T2DM in each CCG and practice) were also able to be estimated.

### Qualitative study findings

#### Feasibility of using ethnographic methods

It was concluded that the methods chosen to generate and collect qualitative data kept the participant burden low and would be feasible in a larger scale RCT. The following sub-sections describe the feasibility of collecting documentary, observational and interview data.

#### Feasibility of collection and review of documents

Both publicly available information and internal communications and documents arising from the intervention delivery were collected. This included emails between the Embedder and stakeholders about local information, planning, refining and evaluating the impact of intervention features; the development of promotional and patient information material; SSME attendance rates; presentations given by the Embedder and providers; meeting notes; and practice workflows. For some of these, further explanation was sought during interviews with the Embedder. These documents helped to understand the wider context of practices, SSME providers and commissioners. Of note, documents revealed the financial, workforce capacity and policy constraints facing practices and providers, which impacted on their ability to improve and innovate patient care.

#### Feasibility of collecting observational data

Over the course of the study, the two qualitative researchers became familiar to practice staff and other stakeholders from regular attendance at meetings and events accompanying the Embedder. It was found to be helpful to spend time engaging in informal conversations with practice staff and observing workflows, use (or non-use) of the intervention tools, as well as interaction with the Embedder. These interactions, and researcher interpretations of them, were documented in detailed narrative field notes. Through more structured observations (for example, shadowing staff during a half-day patient engagement and screening event) further workflow were able to be observed, featuring patient engagement and the availability of education materials, as well as the clinic environment. This approach helped to achieve the goal of gaining a concrete understanding of how a practice works, in order to ground the analyses of the Embedding Package’s adoption within the day-to-day work of practice staff. Observation identified issues that could be fed back to inform refinements to the Embedding Package; for instance, a community engagement event uncovered a previously unidentified potential for patient stigmatisation and highlighted the need to consider the acceptability of locations for public engagement.

#### Feasibility of recruiting to and undertaking individual and group interviews

It was anticipated that it would be possible to arrange face-to-face interviews with stakeholders during lunch breaks or allotted Continuing Professional Development (CPD) or administrative times. However, given constraints on stakeholder availability, more success was had conducting these by telephone. It was helpful to start by briefly recapping their involvement in the study, which enabled us to focus on the most relevant issues. The questions were refined based on the content of the discussion. Once agreeing to participate, no participant withdrew. However, considerable efforts were made by the research team to retain participants including flexibility in the timings, locations and methods of interviewing.

Patient interviews conducted over the telephone at a pre-arranged time were logistically easy, but did not generate useful data, owing to the limited time since the intervention had been implemented, resulting in little impact and change to usual care being reported by patients. This has led to refinement of the plans for the RCT; patients will be recruited and interviewed much later in the implementation process, to allow for the intervention to filter through to patient care.

Interviews with the Embedder transpired to be crucial in facilitating understanding of the embedding process and stakeholder engagement. It became apparent that practice and provider stakeholders were excessively reliant on the Embedder due to resource and capacity limitations, and the need to facilitate low-resource, high-impact strategies was identified. The Embedder’s insights identified the need to build trust and enhance communication with stakeholders to develop their confidence in making process changes; this directly informed the methods and strategies for the RCT.

#### Evaluation of applying Normalisation Process Theory

Overall, NPT was useful in structuring the approach to the ethnographic data collection and analysis. It was a helpful device to sensitise and focus the researchers’ consideration on structural, individual and cognitive factors related to the implementation of the Embedding Package, as well as to uncover challenges and enablers, practical issues and the collection of data (Table 5). Structuring the findings into the NPT domains enabled pragmatic solutions and recommendations to be developed and fed back to the wider team and, in turn, inform the RCT design. However, it was not possible to apply some of the findings to just one particular domain construct and encountered a good deal of crossover between concepts, particularly within the domain of coherence. To enhance the analysis for the RCT, some of the constructs and domain descriptors have been modified to fit the context.

**Table 5:** Normalisation Process Theory constructs, domains and coding examples

Domain (*Components*)	Description	Example
**Coherence** *(differentiation, communal specification, individual specification, internalisation*)	The sense-making work that people undertake individually and collectively	‘One of the practices responded to a very specific question which was around me going and putting the display board up for World Diabetes Day. But when asked to review the Action Plan and things like that have not had any response, and I have followed things up two or three times’ (The Embedder).
**Cognitive participation** (*initiation, enrolment, legitimation, activation*)	The relational work undertaken by people to build and sustain a community of practice around a new intervention	‘No I didn’t use any [Embedding Package Resources] because the doctors don’t actually do the direct referral to [Structured Education] because it is an administrative task’ (GP, Practice 5).
**Collective action***(interactional workability, relational integration, skillset workability, contextual integration)*	The operational work that people undertake to enact a set of practices	‘We sourced a lot of brochures and leaflets from the Diabetes websites and we made contact with a little bit of help from [The Embedder] with the local diabetes support group which has been fantastic they’re really, really, useful’ (Practice Nurse, Practice 5).
**Reflexive monitoring** (*systemisation, communal appraisal, individual appraisal, reconfiguration*)	The appraisal work that people undertake to assess and understand the ways that a new set of practices affects them and others around them	‘In one of the pharmacies that we visited, there was on particular gentleman that has, I don’t know a couple of Pharmacies in the local area, and was really enthusiastic about it, and was giving us feedback as well about people using the Pharmacy, and has given them feedback about [Structured Education] and it was positive’ (Educator, Provider 2).

#### Identifying and collecting contextual data on implementation processes, sustainability of change and fit of the intervention with routine practice

Data identified a number of contextual factors likely to impact on the implementation, adoption and sustainability of the intervention. Leadership, workforce focus and motivation to change were the three main factors identified as impacting intervention implementation and adoption. During the interviews and observations and through document analysis, it became evident that practice leaders had to align innovation work with national and local priorities and areas of focus. Example quotations are referred to in text and are presented in Table 6.

**Table 6:** Example quotations

A	It has been massively beneficial from a personal point of view in terms of helping us get things [monitoring and referral strategies] in place that will hopefully, help longer term. It’s also helped us as an organisation think about things that perhaps we’ve not considered before like working a bit more up closely with Pharmacies and so forth. (Educator Lead)
B	There is a read code that comes in so we can capture the people that have been [to SSME] but really what we want to capture more is the people that have not… And not the ones who have cancelled their appointment, but the ones who didn’t bother to make one even in the first place. (Practice Manager)
C	I have seen plenty of patients walking out with them in various languages and I think sometimes, it is surprising that somebody might have had diabetes for the last 10 years but never really had a good conversation about it. I have had at least one consultation where somebody had read the leaflet and actually, it prompted him or her [patient] to re-engage. I have seen it as a positive thing. (Practice Nurse)
D	We’ve never done anything for World Diabetes Day, Diabetes Awareness anything like that before. Obviously having done several diabetes trials and working with [The Embedder] this year as well, we decided that we would promote it and try and raise awareness. (Practice Nurse)

In relation to leadership, practice managers and lead clinicians regarded it as their responsibility to be both familiar with and committed to the research agenda and improving patient outcomes. Nothing explicit regarding leadership and SSME for T2DM was identified through document analysis of the practices strategy. However, leadership was implicit when organisational issues were described, both at a practice and provider level, as well as wider levels of service commissioning (Table 6; Quotation A).

The focus of the workforce, infrastructure and resource availability were contextual factors that emerged during observations and interviews with practice stakeholders. Challenges in relation to implementation and integration were identified through observations and document analysis and were directly related to the lack of readiness, both in terms of research and the organisation, local infrastructure and limited guidance from commissioners (Table 6; Quotation B).

Motivation to change emerged from the interviews with both practice and provider stakeholders, where participants talked about the potential benefits that could arise from the intervention. Analysis of interviews revealed that practice stakeholders were motivated to use the Embedding Package to inform and refine their practice, if there was a practical and observable benefit for it (Table 6; Quotation C). However, unlike provider participants who reported leadership support to refine existing practices and procedures, practice stakeholders did not address leadership as a focal point when describing how the package was integrated (Table 6; Quotation D). Leadership did, however, emerge as a factor in observational data, but related to a lack of dissemination with respect to the adoption of the intervention in certain practices.

#### Exploring methods to refine the intervention and study procedures

As indicated, a key objective was to develop and optimise methods for feeding back findings from the ethnographic study to the wider team to inform the refinement of the Embedding Package. Bi-monthly meetings were held between two members of the ethnographic team (LH and JT), the Embedder and the trial manager to explore the findings, study challenges and enablers, and any intervention content, potential issues, or study procedures that required further exploration or refinement.

**Fig. 2:**
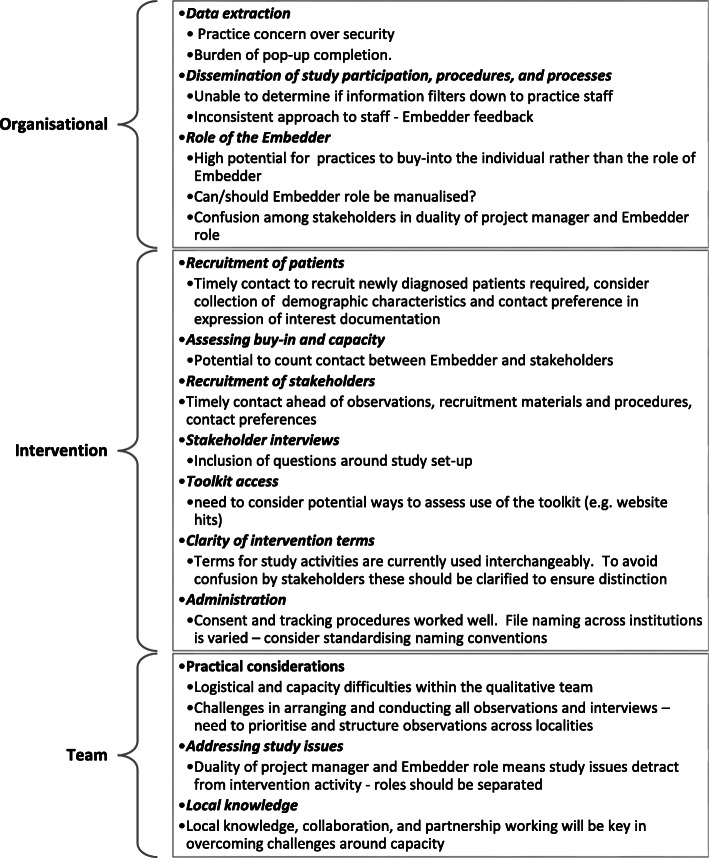
Findings from the ethnographic study, grouped by organisational, intervention and team factors

Findings were shared with the feasibility study team and trial management group on a monthly basis. In brief, findings were structured into three categories (Fig. 2): (a) organisational factors that are specific to the local setting (including support, leadership, culture, values and resources); (b) intervention features that may influence implementation and adoption (e.g. documents, clarity of aims/objectives, systems and procedures, team capacity and feedback); and (c) characteristics of local research teams (including staff roles, communication and information sharing).

Health professionals believed the intervention increased their awareness of SSME, which in turn meant that greater encouragement and more options for accessing SSME could be offered to patients newly diagnosed with T2DM. Case examples demonstrating impact can be found in Additional File 6.

## Discussion

The feasibility of conducting a large-scale evaluation of the Embedding Package in primary care was tested, using a concurrent mixed methods approach. Although no formal feasibility thresholds were set, which is a limitation of this study, we have been able to demonstrate the feasibility of a future RCT based on nine predefined feasibility objectives. A summary of the findings of this study, in relation to each of the feasibility objectives, is provided in Additional File 7.

Data were successfully extracted from primary care health records. Patients were willing to be recruited to the study and to consent to their questionnaire data being linked to their extracted primary care data. It was feasible to capture cost data for Embedding activities and produce a cost estimate for each individual initiative attempted as part of the Embedding Package. Stakeholders from practices that engaged fully reported increased understanding of the content of SSME, which was used to inform the information delivered to patients during consultations. However, there was limited engagement from the majority of the participating practices. Focussing the intervention implementation on practice staff, who are ultimately not responsible for delivering SSME, was not feasible given the capacity constraints in practices. The RCT will therefore focus intervention implementation at the provider-level where possible, whilst maintaining relationships with practices and CCG personnel. This includes action planning meetings taking place with local SSME providers and then cascading the information down to practices when/where appropriate.

Primary care data were extracted for all practices, but from fewer patients than anticipated, as practice sizes were lower than predicted. The proportion of patients returning a completed questionnaire was higher than expected, and over 90% of these then consented to the linking of their data. These recruitment figures will be used to calculate the sample size for the RCT. Generally, there was good completeness of the extracted data, particularly for HbA1c, the primary outcome for the RCT; however, the same cannot be said for the self-reporting of HbA1c. This was over 40% missing; although, where it was reported, it was found to be accurate. This is in line with the findings of research assessing the prevalence of HbA1c self-knowledge among T2DM patients [26]. Due to the poor reporting of HbA1c in the questionnaire, this will be removed from the questionnaire for the RCT.

Other changes will also be made to the questionnaire, including the method of invitation, which will now be solely via postal invite, due to the GP prompts yielding fewer participants and being unpopular with the practices. Biomedical and SSME information and preferences will also be removed from the questionnaire, to better complement the data available from primary care and other studies. Whether or not a participant has been referred to and attended SSME will still be collected, as this has been shown to be a useful tool for validating primary care records. However, there was only moderate agreement between the questionnaire and primary care records in terms of referral and attendance at SSME, suggesting that not all of the discrepancy between the two sources is due to the representativeness of the questionnaire participants. This discrepancy may be due to participants misunderstanding what was meant by SSME; a clearer explanation of what SSME is will therefore be included. There is also the possibility that since referral rates appeared to differ from national figures [10], that some referrals may not have been recorded in the primary care records or that the practices recruited were not as representative as we hoped. The full RCT will include a larger number of practices, so will be more representative. Printed on each questionnaire booklet will be a preassigned participant identification number which can be linked to the individual’s NHS number; this will reduce the quantity of missing NHS numbers which prevented data linkage despite the participant providing consent.

Although it was found to be feasible to collect data for costing the Embedding Package initiatives in the full RCT, it was not feasible to categorise initiative costs based on ‘development’ and ‘steady’ state phases, as these data were planned to be collected by a practice and CCG proforma, but these were not completed. Instead, costs were estimated as an average cost per initiative over the study period. Completing the initiative tracker and proforma by the CCG designates and practice managers proved challenging and unfeasible. It is anticipated that the majority of the work in the RCT will occur at the ‘provider’ level, although most of this may be down to the Embedder. Accordingly, the education provider will be added as a key source for collecting resource use data and the role of the Embedder will be maintained and strengthened in the full RCT. Both CCG designates and practice managers will be removed as sources for collecting resource use data because it has proven unfeasible. Consequently, the resource use data collection tools for the RCT will be amended to reflect this change. Education providers will be supported to ensure that all relevant resource use and cost data are captured over the full RCT follow-up.

The qualitative work highlights that the success of the intervention depends on the organisational context. There were challenges directly related to a lack of readiness, both in terms of research and the organisation, local infrastructure and limited guidance from commissioners. These various contextual factors need to be addressed in the RCT in order to optimise the likelihood for successful implementation and adoption. In order to scale the Embedding Package across many organisations, the work of the Embedder will be imperative to local engagement with and adoption of the intervention. With knowledge of the local context gained at the launch of the intervention and continued contact and development, implementation issues can be identified and mitigated. Furthermore, with the RCT planning to target intervention implementation at the provider level where possible, the challenges identified with practice engagement with the Embedder will be overcome, since one organisation provides SSME for numerous practices [25]. Low levels of uptake and use may be explained in part by a lack of organisational readiness at a local (practice) level and different focus of various stakeholders; thus, aligning interests across multiple stakeholders and organisations remains a challenge when planning an intervention in primary care. Another challenge lies in the implementation process and in the integration of SSME in usual practice. For practices, there is uncertainty about SSME in general, and concerted and ongoing efforts will be required in the RCT to integrate referral and engagement practices as a routine and sustained part of primary care provision. Despite the challenges that were present, a number of low-capacity high-return activities were identified. These include patient information leaflets tailored to the cultural and demographic needs of the practice, promotional videos, waiting room or reception area displays, self-referral forms, and engagement with existing initiatives and partnership working. However, constraints on the timings of this feasibility study meant that the Embedding Package was not utilised sufficiently in practice to have impacted on patient’s awareness and/or experience, particularly at the time patients were recruited to participate in interviews. Patient interviews will be conducted later in the RCT, to allow time for the intervention to have filtered through to patient care. NPT was found to be useful in structuring our approach to ethnographic data collection and analysis. However, it was difficult to apply some of the findings to a single domain construct, and so, to enhance the analysis in the RCT, some of the constructs and domain descriptors will be customised.

## Conclusion

It is feasible to collect demographic, biomedical and SSME referral and attendance data with reasonable completeness and accuracy for the subsequent RCT; however, measures can and will be taken to further improve the quality of the data collected. Based on this feasibility study, it is also feasible to collect data for costing the Embedding Package initiatives for the full RCT; however, changes are required to some elements of the resource use data collection strategy. Based on data collected, it was feasible to produce cost estimates for each individual initiative attempted as part of the Embedding Package. The methods chosen to generate, collect and analyse qualitative data were satisfactory; they kept participant burden low and provided insight into potential refinements of the Embedding Package. Some further development of the Embedding Package is required to improve engagement from practices.

## Supplementary information

**Additional file 1.** Ethnographic interview topic guides.

**Additional file 2.** Unit costs applied for valuation of resource use.

**Additional file 3.** Summary of extracted continuous primary care data by practice.

**Additional file 4.** Other reasons given for not attending SSME.

**Additional file 5.** Other reasons given for not wanting to attend SSME if invited.

**Additional file 6.** Case examples demonstrating impact.

**Additional file 7.** Summary of the findings for each feasibility objective.

## Data Availability

The dataset used and analysed during the current study is available from the corresponding author on reasonable request.

## Abbreviations

BMI: Body mass index
CCG: Clinical commissioning group
CPD: Continuing Professional Development
DBP: Diastolic blood pressure
DNA: Did not attend
GP: General practitioner
HDL: High-density lipoproteins
NHS: National Health Service
NICE: National Institute for Health and Care Excellence
NPT: Normalisation Process Theory
RCT: Randomised controlled trial
SBP: Systolic blood pressure
SD: Standard deviation
SSME: Structured self-management education
T2DM: Type 2 diabetes mellitus
UK: United Kingdom
