# Supplementary material for: Increasing uptake of structured self-management education programmes for type 2 diabetes in a primary care setting: a feasibility study
Source: Pilot Feasibility Stud. 2020 May 22;6:71. doi: 10.1186/s40814-020-00606-0 (PMC7243310; doi:10.1186/s40814-020-00606-0)
Supplement: Supplementary file 1 — Additional file 1. Ethnographic interview topic guides. [file 40814_2020_606_MOESM1_ESM.pdf]

Appendix 1: Ethnographic interview topic guides

1. Interviews with Practice Staff

Introductory questions:

a) Can you tell me your job role and how this relates to the care of patients with T2D / delivery of diabetes education?
b) Which structured education (SE) programme(s) are your patients in your practice with T2D referred to?
c) What have been the challenges to referral and/or uptake of [this SE programme] to date?
    - *Prompts:* what do you think has impeded / helped this?

Questions:

a) Over the last X months an 'embedding package' has been trialled in this CCG.
    I. Can you tell me what you know about this / what it involves?
b) Questions relating to:
    I. A marketing strategy – (Q: What marketing have you seen?)
    II. Improved referral & data collection processes – (Q: can you tell me how anything has changed in the way that you refer people to [SE/the programme]? What about changes to the data that is collected about SE referral/uptake?)
    III. A local champion - Q: Are you aware of a local champion for SE? What contact have you had with them? How important is the role?
    IV. An 'Embedder' - Q: Are you aware of this person and their role? What contact have you had with them? How important is the role?
    V. The administrator of the SE programme (i.e. the person who arranges the courses and books people on to them) – Q: Have you had any contact with them? How helpful was that? How important is the role?
    VI. A tool kit (containing practical advice and resources) – (Q: Are you aware of a tool kit relating to SE? Have you used the tool kit? If so, how? Which tools have you used? How was it helpful? How could it have been more helpful?)

c) How do you think the embedding package has addressed / will address the challenges to referral/uptake that you mentioned earlier?
    I. Which aspects do you think have been most/least helpful and why? / What worked well/didn't work well and why
        - Is there anything missing/not needed and why?
        - Has it changed/improved anything? How? E.g.:
        - Uptake to SE,
        - Outcomes for the participants?
        - How SE is received by patients?
        - A difference to how you work?
    II. Can you see it working long term? Will all the elements always be needed (e.g. marketing, the champion)?
d) In terms of SE, what do you think about the option of an online course?

2. ***Interviews with Patients***

Introductory questions:

a) Demographic data (see appendix)
b) Would you mind telling me your age? How you would describe your ethnicity? Do you have a disability?
c) How long have you been diagnosed with T2D?
d) What were you told about diabetes when you first found out you had it? What information or advice were you given?

Questions:

a) Tell me a bit about your experience with diabetes education since you've been diagnosed?
b) What about your experience with [name of SE programme]:
   I. How did you get involved with it? (Who told you about it? Were you referred? By whom?)
   II. How were your needs and preferences taken into account as part of this referral?
   III. What were your expectations?
   IV. What did you like about it? What would you change?
   V. Would you refer a family member or friend?
   VI. What makes it easier / harder to attend?
   VII. Do you think it would suit everyone or are there certain people it wouldn't suit?
   VIII. Would you like to have the option of an online course? Instead of/as well as what you have now?
   IX. What about other ways to get this information/education?
   X. What should we call these kinds of programmes? is 'diabetes education' the right name?
c) Over the past [number of months] months, your practice has been trying improve the way it identifies and refers people for help with managing their diabetes and to make sure that [SE programme] meet people's needs…
   I. How has your experience during these X months differed from previous diabetes care / education?
   II. What was done well?
   III. What could have been improved?

3. INTERVIEW WITH STRUCTURED EDUCATION DELIVERERS/PROVIDERS

Introductory questions:

a) Can you tell me your job role and how this relates to the delivery of diabetes education?

b) Which structured education (SE) programme(s) do you deliver/provide?
c) What challenges have you been aware of in the referral and/or uptake of [this SE programme] to date?

- what do you think has impeded / helped this?

Questions:

a) Over the last X months an 'embedding package' has been trialled in this CCG.
   I. Can you tell me what you know about this / what it involves?
b) Questions relating to:
   I. A marketing strategy – (Q: What marketing have you seen?)
   II. Improved referral & data collection processes – (Q: can you tell me how anything has changed in the way that people are referred to [this SE programme]? What about changes to the data that is collected about SE referral/uptake?)
   III. The administrator of the SE programme (i.e. the person who arranges the courses and books people on to them) – Q: Can you talk about how you work with them? How helpful is this process? How important is their role?
   IV. A local champion - Q: Are you aware of a local champion for SE? What contact have you had with them? How important is the role?
   V. An 'Embedder' - Q: Are you aware of this person and their role? What contact have you had with them? How important is the role?
   VI. A tool kit (containing practical advice and resources) – (Q: Are you aware of a tool kit relating to SE? Have you – or anyone you work with – used the tool kit? If so, how? Which tools were used? How was it helpful? How could it have been more helpful?)

b) How do you think the embedding package has addressed / will address the challenges to referral/uptake that you mentioned earlier?
   I. Which aspects do you think have been most/least helpful and why? / What worked well/didn't work well and why
   II. Is there anything missing/not needed and why?
   III. Has it changed/improved anything? How? E.g.:
   IV. Uptake to SE,
   V. Outcomes for the participants?
   VI. How SE is received by patients?
   VII. A difference to how you work?
   VIII. Can you see it working long term? Will all the elements always be needed e.g. marketing, the champion?

c) Is there an online version of this SE programme? Can you tell me about that?
d) What do you think about the option of an online course? What are the pros and cons?

4. ***Interviews with Commissioners***

Introductory questions:

a) Can you tell me a little bit about your job/role (also prompt re how big CCG region is etc.)
b) Have you been involved in the commissioning of structured education programmes for self-managing chronic disease (specifically diabetes?)
c) Can you tell me about the most recent time that you've commissioned a programme? Can you tell me more about the [commissioning /decision-making] process? What was important to consider when making that decision?
d) Have you considered/would you consider commissioning an online course?
e) What relationship do you have with the providers? Do you meet with them/monitor them regularly?
f) Would you find this feedback useful? How would it impact future commissioning decisions?
g) How would you like to receive this information?

Questions:

a) Over the last X months an 'embedding package' has been trialled in this CCG.
    I. Can you tell me what you know about this / what it involves?

b) Questions regarding:
    I. A marketing strategy – (Q: What marketing have you seen?)
    II. Improved referral & data collection processes – (Q: can you tell me how anything has changed in the way that people are referred to [SE/the programme]? What about changes to the data that is collected about SE referral/uptake?)
    III. A local champion - Q: Are you aware of a local champion for SE? What contact have you had with them? How important is the role?
    IV. An 'Embedder' - Q: Are you aware of this person and their role? What contact have you had with them? How important is the role?
    V. The administrator of the SE programme (i.e. the person who arranges the courses and books people on to them) – Q: Have you had any contact with them? How helpful was that? How important is the role?
    VI. A tool kit (containing practical advice and resources) – (Q: Are you aware of a tool kit relating to SE? Have you used the tool kit? If so, how? Which tools have you used? How was it helpful? How could it have been more helpful?)

c) Which elements do you think that the provider is responsible for delivering and/or which are the responsibility of the CCG?
d) How do you think such an Embedding Package might improve and sustain uptake?
e) What outcomes would you expect to see from the use of this tool kit?
