# Supplementary material for: Increasing uptake of structured self-management education programmes for type 2 diabetes in a primary care setting: a feasibility study
Source: Pilot Feasibility Stud. 2020 May 22;6:71. doi: 10.1186/s40814-020-00606-0 (PMC7243310; doi:10.1186/s40814-020-00606-0)
Supplement: Supplementary file 2 — Additional file 2. Unit costs applied for valuation of resource use. [file 40814_2020_606_MOESM2_ESM.pdf]

Appendix 2: Unit costs applied for valuation of resource use

Description	Unit cost	Source	Note
Embedder (per hour	£42	PSSRU 2016[1]	NHS agenda for change band 6
Diabetes Lead (per hour)	£25	PSSRU 2016	Band 3
Administrator (per hour)	£25	PSSRU 2016	Band 4
General Practitioner (GP) per hour	£134	PSSRU 2016	With qualification (excluding direct care staff cost)
Research staff (per hour)	£42	PSSRU 2016	Band 6
GP Nurse (per hour)	£43	PSSRU 2016	Band 7
Research Nurse (per hour)	£52	PSSRU 2016	Band 7
Public Health Worker (per hour)	£32	PSSRU 2016	Band 5
Diabetes Educator (per hour)	£25	PSSRU 2016	Band 3
Voluntary service worker (per hour)	£11	UK ASHE 2015[2]	-
Practice Manager (per hour)	£73	PSSRU 2016	Band 8b

[1] Curtis L, Burns A. Unit Costs of Health and Social Care 2016. Canterbury: PSSRU, University of Kent; 2016. URL: www.pssru.ac.uk/project-pages/unit-costs/2016/ (accessed 11 Dec 2017)

[2] UK Annual Survey of Hours and Earnings (ASHE) 2015. URL: https://www.wcva.org.uk/media/5625220/10_volunteer_time_as_match_funding.pdf (accessed 11 Dec 2017)
