# Supplementary material for: Increasing uptake of structured self-management education programmes for type 2 diabetes in a primary care setting: a feasibility study
Source: Pilot Feasibility Stud. 2020 May 22;6:71. doi: 10.1186/s40814-020-00606-0 (PMC7243310; doi:10.1186/s40814-020-00606-0)
Supplement: Supplementary file 3 — Additional file 3. Summary of extracted continuous primary care data by practice. [file 40814_2020_606_MOESM3_ESM.pdf]

Variable	CCG 1						CCG 2					
	Practice 1		Practice 2		Practice 3		Practice 4		Practice 5		Practice 6	
	% missing	Mean (SD)	% missing	Mean (SD)	% missing	Mean (SD)	% missing	Mean (SD)	% missing	Mean (SD)	% missing	Mean (SD)
Age, *years*	0.0	69.2 (12.0)	0.0	68.6 (12.8)	0.0	66.0 (13.7)	0.0	65.6 (13.1)	0.0	63.9 (13.9)	0.0	64.3 (13.0)
HbA1c, *mmol/mol*	1.8	55.5 (13.0)	2.5	54.2 (13.2)	2.5	55.0 (15.5)	6.2	53.9 (14.5)	10.0	60.1 (17.5)	13.2	55.0 (17.3)
Weight, *kg*	6.5	87.2 (20.2)	11.1	83.3 (19.7)	11.0	87.3 (22.9)	13.2	92.0 (23.4)	35.0	88.1 (22.5)	48.1	86.8 (20.6)
Total cholesterol, *mmol/L*	3.9	4.3 (1.1)	5.6	4.2 (1.2)	21.2	4.3 (1.0)	9.0	4.2 (1.0)	17.5	4.4 (1.1)	23.6	4.3 (0.9)
HDL cholesterol, *mmol/L*	4.7	1.3 (0.3)	6.1	1.3 (0.3)	21.2	1.3 (0.4)	11.0	1.3 (0.4)	20.1	1.3 (0.4)	53.8	1.4 (0.5)
Systolic blood pressure, *mmHg*	2.5	133.3 (14.6)	5.0	133.4 (13.0)	7.6	136.2 (11.8)	8.2	131.0 (13.0)	9.2	131.6 (14.4)	9.4	133.9 (12.8)
Diastolic blood pressure, *mmHg*	2.5	74.8 (9.6)	5.0	74.7 (9.7)	7.6	78.9 (8.5)	8.2	74.2 (9.8)	9.2	74.7 (9.3)	9.4	77.8 (8.5)
QRisk Score, *%*	69.4	25.5 (14.6)	70.6	23.1 (15.3)	23.7	27.9 (16.6)	34.9	28.3 (15.9)	33.9	17.4 (13.2)	86.8	20.3 (11.3)
	**Percentage**											
Sex												
Male		54.1		56.7		58.5		55.8		54.1		55.7
Female		45.9		43.3		41.5		44.2		45.9		44.3
Ethnicity												
White European		54.8		64.4		67.8		85.3		72.2		43.7
South Asian		0.5		10.8		11.9		2.0		14.4		9.4
Black		0.2		0.3		2.5		1.1		4.7		1.6
Other		0.5		1.7		2.5		0.6		1.2		1.9
Not otherwise stated		18.1		0.0		0.9		0.9		1.2		0.9
Missing		25.8		22.8		14.4		10.2		6.2		42.5

Smoking Status						
Never smoker	48.6	50.6	46.6	46.4	49.9	57.2
Ex-smoker	41.8	41.4	44.9	40.2	33.7	34.0
Current smoker	9.7	8.1	8.5	13.5	16.4	8.8
HbA1c, mmol/mol						
≤53 (≤7%)	54.1	61.1	53.4	56.9	39.8	48.4
54-58 (7.1-7.5%)	14.3	14.4	13.6	11.6	14.3	12.0
59-64 (7.6-8.0%)	10.6	8.1	10.2	9.9	10.3	7.9
65-69 (8.1-8.5%)	7.2	3.3	5.1	5.2	5.0	5.4
70-86 (8.6-10.0%)	8.6	6.1	11.0	6.6	13.2	8.8
≥87 (≥10.1%)	3.4	4.4	4.2	3.7	7.4	4.4
Missing	1.8	2.5	2.5	6.2	10.0	13.2
Diabetes Medication in Last 12 Months						
None	19.9	24.4	13.6	26.5	18.3	27.7
DPP-IV	2.7	1.7	0.9	2.3	5.8	4.7
GLP-I	1.3	0.8	0.0	0.6	0.6	0.3
Insulin	9.0	6.7	5.1	9.6	10.8	8.5
Metformin	52.9	53.3	61.0	48.2	48.9	39.6
SGLT-2	0.9	0.0	0.0	1.0	0.7	0.9
Sulphonylurea	12.4	1.2	19.5	9.9	13.4	9.8
Multiple medications	0.0	0.0	0.0	1.0	1.2	6.6
Other	1.1	0.8	0.0	1.0	0.2	1.9
Previous Atrial Fibrillation Diagnosis						
Yes	8.6	13.1	9.3	10.3	5.5	7.2
No	91.4	86.9	90.7	89.7	94.5	92.8
SME Referral						
No record	57.2	41.4	66.1	64.8	68.0	78.6
DESMOND	7.2	12.2	18.6	32.9	26.0	11.3
X-PERT	0.0	0.0	0.0	0.0	0.2	0.0
DAFNE	0.4	0.0	0.0	1.3	0.7	0.3
Referred but course not recorded	35.3	46.4	15.3	1.0	5.1	9.8

Date of SME Referral						
≤1 year	7.2	18.1	28.0	9.3	7.0	2.8
>1 year	35.7	40.6	5.9	25.9	25.0	18.6
Missing	57.2	41.4	66.1	64.8	68.0	78.6
SME Attendance						
Not referred	35.8	20.3	32.2	57.7	47.8	65.7
Attendance not recorded	25.6	28.1	18.6	7.2	7.3	8.5
Did not attend	30.1	48.1	45.8	18.0	23.2	10.1
Attended	8.4	3.6	3.4	17.2	21.7	15.7
Date of SME Attendance						
≤1 year	2.0	0.0	0.9	5.7	5.8	6.3
>1 year	6.5	3.6	2.5	11.3	15.9	9.1
Missing	91.6	96.4	96.6	83.0	78.3	84.3
