# Supplementary material for: Increasing uptake of structured self-management education programmes for type 2 diabetes in a primary care setting: a feasibility study
Source: Pilot Feasibility Stud. 2020 May 22;6:71. doi: 10.1186/s40814-020-00606-0 (PMC7243310; doi:10.1186/s40814-020-00606-0)
Supplement: Supplementary file 4 — Additional file 4. Other reasons given for not attending SSME. [file 40814_2020_606_MOESM4_ESM.pdf]

Didn’t like group format

- All I'm interested in is precise information about the state of my health. I do not wish for group therapy or to listen to competitive ailment conversations. A low key approach to diabetes suits me well but I do wish for more monitoring.
- I am not good in group situations.
- I would prefer my conversation to be private but appreciate a group may be financially desirable.
- Not interested in groups.

Couldn’t book onto a course

- At the time of me applying to join the DESMOND programme in [place name omitted] they were having problems in organising the courses so I never got the opportunity to attend.
- I phoned several times but always only got an answer machine. I never got to speak to anyone and had been diagnosed at least 10 years by this time anyway.

Condition being satisfactorily managed/OK on own

- Because the practice nurse said my urine sample was fine and my blood sample results were fine and they did not tell me they took me off the Type 2 diabetes tablets ie I didn't need them.
- Diabetes - marginal
- I appear to be making satisfactory progress with my own dieting and exercise regime.
- I manage my condition without a need for a diabetes education programme.
- I no longer have diabetes as I lost weight and followed a diet.
- It was manageable by changing diet.
- My and I feel we know what to do and keep ourselves well informed (eg from the web and reading). Diet, weight and exercise are all considered all the time!.
- My knowledge already quite good. I was a Nursing Lecturer.

Barriers to getting to SME

- Cannot drive, sick husband, working hours.
- 1. I can't walk, virtually housebound with osteoarthritis. 2. Couldn't give a turkeys.
- It was not in my village so I unable to attend.
- Too far away.

Language/communication barriers to attending SME

- I don't speak the language and can't understand English.
- I speak only Polish language if you give me an interpreter I will go on sessions. I will prefer then online sessions with no interpreter.
- Language problem. My first language is Gujarati.
- Learning disability, bi-polar and autism sometimes affect my ability in social settings
- On very basic understanding of English.
- Need it to be in Gujarati for me to attend. English is my second language.

Not invited

- I have put no, but I really cannot remember that far back if I was asked or not - but I would have gone I'm sure, but my Diabetes Nurse has talked about my diabetes.

- In Q8 I recorded that I had never been invited and in Q10 I recorded that I had never attended it therefore seems pointless to ask if I was ever re-invited, therefore? I WAS NEVER INVITED!
- Not been asked to.
- Not given the option of a group session.
- Not invited.

Other

- Did not want to.
- Disappointed with attitude of nurse suggesting unrealistic changes to lifestyle eg give up your job.
- Do not know of any.
- Love to learn more.
