# Supplementary material for: Increasing uptake of structured self-management education programmes for type 2 diabetes in a primary care setting: a feasibility study
Source: Pilot Feasibility Stud. 2020 May 22;6:71. doi: 10.1186/s40814-020-00606-0 (PMC7243310; doi:10.1186/s40814-020-00606-0)
Supplement: Supplementary file 5 — Additional file 5. Other reasons given for not wanting to attend SSME if invited. [file 40814_2020_606_MOESM5_ESM.pdf]

Condition managed sufficiently

- After 10 years everything going well.
- Because I am now off all medication.
- Diabetes monitored at surgery.
- Don't need one. Blood pressure and levels monitored by me.
- GP care is thorough and sufficient.
- I believe my diabetes is under control.
- I control my diabetes by reducing carbs. Was not taught this way.
- I keep my diet under control and I'm very active.
- I know how to manage it now.
- Managing ok
- Not needed if things deteriorate I would be happy to.

Satisfied with current level of knowledge

- Already familiar with information offered
- I am very au fait with condition.
- I feel I understand and can manage the disease. I was a district nurse for 25 years until retirement 2005.
- I get sufficient information from my annual check-ups.
- I think my knowledge still good and searching skills ok.
- Think I have taken everything on board.
- Prefer to research myself.

Already attended a course

- Already attended Desmond.
- Already attended a session
- Already attended programme
- Already done DESMOND.
- Already done so.
- Juggle was excellent in clearly explaining the way diabetes worked. It’s my 'gannet mentality' that needs adjusting.
- The Juggle programme gave me all the information.

Barriers to getting to SME

- Anything outside home difficult to suit me.
- Can't walk. Don't give a turkeys.
- Cannot drive, sick husband, working hours.
- Difficulty in travelling
- Don't go out.
- Not sure depends on location.
- Only if it was in my village.
- Work commitments.

Other medical condition

- Alzheimer's - wife is full time carer.

- I'm disabled

Language barrier

- Don't speak the language (don’t understand).
- Language barrier and able to control by myself.
- Poor English language

Would like to see changes to course

- 8 hours of Desmond course could have been condensed to 1 hour.
- Would attend if it were very different to Desmond.
- I am waiting for you to change the guidelines to follow a low carb lifestyle for diabetes, where the HbA1c, BP and other inflammatory markers are reduced.

Other

- Not interested.
- Too old.
- Not necessary
