# Supplementary material for: Increasing uptake of structured self-management education programmes for type 2 diabetes in a primary care setting: a feasibility study
Source: Pilot Feasibility Stud. 2020 May 22;6:71. doi: 10.1186/s40814-020-00606-0 (PMC7243310; doi:10.1186/s40814-020-00606-0)
Supplement: Supplementary file 6 — Additional file 6. Case examples demonstrating impact. [file 40814_2020_606_MOESM6_ESM.pdf]

**Educator impact**
Utilising the tool-kit and with guidance from the Embedder, education providers piloted patient self-referral, developed and refined a referral form for use by healthcare professionals and patients, currently being rolled-out across the county, and networking with community pharmacies to promote self-referral and attendance at diabetes education. To celebrate World Diabetes Day, educators organised a community event to engage with the general public and pharmacies, providing general information on diabetes and DESMOND courses:<br>"*I think it's been massively beneficial from a personal point of view in terms of helping us get things in place that will hopefully help longer term. It's also helped us think about things that perhaps we've not considered before like working a bit more up closely with Pharmacies and so forth, and also at the wider community*". (Educator, Provider 2).

**Practice impact**
Utilising the Embedding package and guided by the Embedder a detailed Action Plan was developed. As part of the Action Plan, healthcare professionals engaged with the opportunity to receive information about local structured education programmes and pose questions concerning the delivery and content of such sessions. General diabetes literature suitable for their patient population (e.g. information in other languages and culturally specific literature) and text and promotional videos were displayed in the practice waiting areas to increase patient awareness. Self-referral sheets resembling prescription forms (FP10s) were introduced in the practice to reiterate to patients that structured education is an effective 'treatment' option. The practice also participated in a range of activities to celebrate World Diabetes Day. These activities included a range of diabetes related displays, promotion of structured education, and a collaborative diabetes screening event with the practice's PPG group:<br>"We sent a text out to patients that basically said that [the practice] was supporting World Diabetes Week and we gave the date of the World Diabetes Day and that we would be offering information, advice and free screening… and anything that came up abnormal on any of the screening that we did we would then raise with their own GP afterwards". (Practice Nurse, Practice 5).
