# Supplementary material for: Increasing uptake of structured self-management education programmes for type 2 diabetes in a primary care setting: a feasibility study
Source: Pilot Feasibility Stud. 2020 May 22;6:71. doi: 10.1186/s40814-020-00606-0 (PMC7243310; doi:10.1186/s40814-020-00606-0)
Supplement: Supplementary file 7 — Additional file 7. Summary of the findings for each feasibility objective. [file 40814_2020_606_MOESM7_ESM.pdf]

Appendix 7: Summary of the findings for each feasibility objective

Objective	Summary of findings
**Quantitative**	
1. Assess the feasibility of two recruitment and consent approaches	• 85% of patients were recruited by postal invite and 15% by GP prompt.<br>• GP prompts were unpopular with the practices.
2. Assess the feasibility of extracting demographic and biomedical data, and information on SSME referral and attendance from primary care medical records with sufficient accuracy and completeness for use in an RCT, particularly HbA1c, the primary outcome for the RCT	• There was consistent accuracy across CCGs and practices.<br>• HbA1c was less than 10% missing and most data was at least 80% complete. However, BMI and hospital admissions was missing for all patients.<br>• Some large differences in data completeness were driven by differences between practices.
3. Assess patient willingness to provide consent for accessing and extracting identifiable data from their medical records	• 90.8% of patients consented to their questionnaire and primary care data being linked.
4. Assess the willingness of patients to provide consent and complete a questionnaire asking for demographic data and information on the diagnosis and management of their diabetes, their history of being invited to or attending SSME and their preferences around the method of delivery of SSME	• 14.7% of patients completed the questionnaire.<br>• Self-reported HbA1c was missing for 42% of patients, but accurate.<br>• SSME referral and attendance was well reported, but there was only moderate agreement with primary care records.
5. Assess the feasibility of capturing cost data for embedding activities at participating practices and Clinical Commissioning Groups (CCGs)	• Average cost estimates per initiative over the study were calculated based on the Embedder's tracker.<br>• Initiative costs couldn't be categorised based on 'development' and 'steady' state phases as none of the CCGs designates or practice managers completed the intervention tracker or proforma.
**Qualitative**	
1. Assess the feasibility of using ethnographic methods in a range of primary care settings	• The ethnographic methods used kept the participant burden low and would be feasible in a larger scale RCT.
2. Identify and collect context-specific data on the processes of implementation, sustainability of change, and the fit of the Embedding Package with routine practice	• Leadership, workforce focus and motivation to change were the main factors impacting intervention implementation and adoption.<br>• There was a lack of dissemination with respect to the adoption of the intervention in certain practices. There were also challenges directly related to the lack of readiness of research and the organisation, local infrastructure and limited guidance from commissioners. Practice stakeholders were motivated to use the Embedding Package to inform and refine their practice, if there was a practical and observable benefit for them.
3. Evaluate the application of Normalisation Process Theory (NPT) as a way of analysing data and understanding factors contributing to the embedding of SSME	• NPT was useful in structuring the approach to the ethnographic data collection and analysis.<br>• However, it was difficult to apply some of the findings to a single domain construct.
4. Explore methods for providing formative feedback to aid in the refinement of the Embedding Package and development of RCT study procedures	• Some low capacity-high return activities were identified and fed back, these included: tailored patient information leaflets; promotional videos; waiting room displays; self-referral forms; and engagement with existing initiatives and partnership working.
